# Short-term triple therapy with azithromycin for *Helicobacter pylori *eradication: Low cost, high compliance, but low efficacy

**DOI:** 10.1186/1471-230X-8-20

**Published:** 2008-05-29

**Authors:** Fernando M Silva, Jaime N Eisig, Ana Cristina S Teixeira, Ricardo C Barbuti, Tomás Navarro-Rodriguez, Rejane Mattar

**Affiliations:** 1 Serviço de Gastroenterologia Clínica, Hospital das Clínicas da Faculdade de Medicina da Universidade de São Paulo, Av. Dr. Enéas de Carvalho Aguiar, 155, 9°. Andar, Cerqueira Cezar, São Paulo, SP, CEP: 05 403-900, Brasil

## Abstract

**Background:**

The Brazilian consensus recommends a short-term treatment course with clarithromycin, amoxicillin and proton-pump inhibitor for the eradication of *Helicobacter pylori *(*H. pylori)*. This treatment course has good efficacy, but cannot be afforded by a large part of the population. Azithromycin, amoxicillin and omeprazole are subsidized, for several aims, by the Brazilian federal government. Therefore, a short-term treatment course that uses these drugs is a low-cost one, but its efficacy regarding the bacterium eradication is yet to be demonstrated. The study's purpose was to verify the efficacy of *H. pylori *eradication in infected patients who presented peptic ulcer disease, using the association of azithromycin, amoxicillin and omeprazole.

**Methods:**

Sixty patients with peptic ulcer diagnosed by upper digestive endoscopy and *H. pylori *infection documented by rapid urease test, histological analysis and urea breath test were treated for six days with a combination of azithromycin 500 mg and omeprazole 20 mg, in a single daily dose, associated with amoxicillin 500 mg 3 times a day. The eradication control was carried out 12 weeks after the treatment by means of the same diagnostic tests. The eradication rates were calculated with 95% confidence interval.

**Results:**

The eradication rate was 38% per intention to treat and 41% per protocol. Few adverse effects were observed and treatment compliance was high.

**Conclusion:**

Despite its low cost and high compliance, the low eradication rate does not allow the recommendation of the triple therapy with azithromycin as an adequate treatment for *H. pylori *infection.

## Background

The eradication of *Helicobacter pylori *(*H. pylori*) in the treatment of peptic ulcer is currently a world consensus [1-6].

Several therapy courses have been employed in the eradication of the bacterium, with the use of drugs such as bismuth, clarithromycin, amoxicillin, furazolidone, nitroimidazole compounds and proton pump inhibitors, in assorted combinations [7-11].

The search for low-cost and higher efficacy with fewer adverse effects, that can allow higher compliance to *H. pylori *eradication therapy, is a vital concern [12-16]. In Brazil, where several particular situations are present, such as higher bacterial resistance to antibiotics [17,18], special health conditions [19] and low socioeconomic status of the population [20,21], this task is even more delicate.

The macrolides are among the antibiotics that used alone, present high rates of bacterium eradication, with few adverse effects and simple regimen, especially clarithromycin [22,23], although its previous use, similarly to what occurs with nitroimidazole drugs, can determine secondary bacterial resistance [24].

In Brazil, the triple therapy with amoxicillin and clarithromycin, associated to a proton pump inhibitor for 7 days, has attained good eradication rates [25], possibly because this therapy has high compliance and presents low bacterial resistance [18,26-28]. Hence, it has been recommended as a first choice treatment in a national consensus [5], although with a high cost (around US$75.00).

Azithromycin, a macrolide with a long term action [29,30], is part of the assortment of drugs available for *H. pylori *treatment [31-34]. However, some studies have shown low eradication rates [35,36]. In our country, a study has shown good efficacy of this antibiotic when associated with furazolidone [37]. Although another study of our group, associating azithromycin with secnidazole in an ultra-fast treatment course, presented low efficacy [38].

The Public Health services in our country do not provide any eradication treatment for *H. pylori*, free of charge. However, the federal government subsidizes, among other drugs, the acquisition of omeprazole, azithromycin and amoxicillin, through its Popular Pharmacy program [39]. With the objective of offering a low-cost treatment to eradicate the bacterium (in this case, at a cost of US$10.00) we tested a 6-day drug therapy with azithromycin associated with amoxicillin and omeprazole.

## Methods

### Setting

Sixty outpatients with *H. pylori *positive peptic ulcer, followed at the Service of Gastroenterology of Clinics Hospital of the Medical School, University of São Paulo were randomly invited to participate in the study. All patients signed a free and informed consent form prior to enrollment. The study was approved by the Ethics Committee of the hospital.

### Inclusion and exclusion criteria

The inclusion criteria were: peptic ulcer diagnosed by upper digestive endoscopy, and *H. pylori *infection, confirmed by rapid urease test, histological analysis and the Urea Breath Test (UBT).

The exclusion criteria were: age younger than 16 or older than 90 years, chronic use of acetylsalicylic even at low doses, or other anti-inflammatory drugs, previous use of macrolides, use of antibiotics or chemotherapeutic drugs in the 4 weeks prior to study enrollment, having complicated peptic ulcer, pyloric stenosis, previous gastric surgery, erosive esophagitis, to be pregnant or breastfeeding, having consumptive diseases or not controlled renal, heart or hepatic failure, having been previously treated for *H. pylori *eradication or having participated in any other clinical studies in the two months prior to the study enrollment.

### Study design

The eradication treatment was carried out with omeprazole 20 mg and azithromycin 500 mg in a single daily dose taken in fasting condition in the morning, associated with amoxicillin 500 mg, taken three times a day immediately after meals, for six days.

The adverse effects and the compliance were documented on the first day after the end of the treatment. The type, intensity and duration of the adverse effects were recorded. The medication blisters were assessed and the remaining tablets or capsules were counted.

Treatment control was carried out 10 to 12 weeks after the end of treatment, when the UBT and an upper digestive endoscopy done. Samples from corpus and antrum mucosa were colleted to perform the rapid urease test and histological analysis.

Patients were considered cured when they presented negative results in at least two of the performed tests and, in case of discordant test results, a new UBT was performed two months after the control.

The symptomatic patients were allowed to use antacid medication after the drug therapy up to the time when the eradication control tests were performed.

### Statistical analysis

The statistical analysis was carried out with the SPSS program, version 10.0 (SPSS Inc. USA).

The sample calculation was determined by means of a descriptive study of one dichotomous variable, in which the prevalence of peptic ulcer disease in the general population was assumed to be 8% and the lower bacterium eradication efficacy was 75%. The eradication rates were studied per intention to treat and per protocol. All of the patients included in the study were considered for the analysis of per intention to treat. The patients who took most of the medication adequately and came back for the control evaluation were considered for the per protocol analysis. The 95% confidence interval was determined for the eradication rates.

## Results

The study group consisted of sixty patients. The characteristics of the studied population are shown in Table 1.

**Table 1:** Characteristics of the studied population.

Patients		60
Age (yrs)	Mean	48
	Median	47
	Range	21 – 83
Sex	Women	31 (52%)
	Men	29 (48%)
Peptic ulcer risk	Smokers	21 (35%)
	NSAIDs* users	16 (27%)
Peptic ulcer type	Gastric	24 (40%)
	Duodenal	31 (52%)
	Gastric + Duodenal	5 (8%)

NSAIDs* = Non-Steroidal Anti-Inflammatory Drugs

Duodenal ulcers were present in 52% of the patients. Cigarette smoking was reported by 35%. Four patients not return for the eradication control. One of these patients withdrew the medication after only two days, due to the presence of diarrhea. Adverse effects were reported by 20 of the 60 patients included in study (33%), being considered mild by 17 patients (28%), moderate by two patients (3%) and severe by only one patient, who withdrew the treatment. The most frequent adverse effects were diarrhea (22%) and nausea (5%).

The bacterium eradication was attained by 23 of the 56 patients assessed: 41% per protocol (Table 2).

**Table 2:** Eradication rates.

	Rate and percentage	Confidence interval (95%)
	
Intention to treat	23/60 38%	51 – 26%
Per Protocol	23/56 41%	54 – 28%

## Discussion

In Brazil, the majority of the population depends on public health services to have access to healthcare [40]. In addition, part of the population that has private health insurance or can afford private healthcare services, depends on the government's subsidy or free medication programs to obtain the drugs. Some healthcare programs such as the tuberculosis [41], AIDS [42], as well as the diabetes or hypertension [43] warrant free medication to all of the Brazilian population. Although it is a consensus that the curative treatment of peptic ulcer disease depends on the eradication of the *H. pylori *[44] and that the cost benefit ratio is favorable regarding this approach [45], the government does not provide any eradication strategy free of charge through the public health system.

The country has characteristics of a developing country, regarding the aspect of income distribution [20,21], as well as the prevalence (quite high) of *H. pylori *infection [46-49], with the bacterium being resistant to many antibiotics [18]. However, the treatment with a proton pump inhibitor, amoxicillin and clarithromycin has reached good eradication rates [25], close to those observed in developed countries [50] and differently from those observed with the association of proton pump inhibitor, nitroimidazoles and clarithromycin [51,52], possibly due to the primary resistance of *H. pylori *to these compounds [17,18]. There are different packs of triple treatments in the Brazilian market, associating a proton pump inhibitor, amoxicillin and clarithromycin, presented in blisters for daily use during 7 days of treatment, which favors treatment compliance and control [53,54]. However, due to its cost, it cannot be afforded by the majority of the population.

In Brazil, peptic ulcer disease and gastric cancer represent important causes of mortality [55,56] and thus, the eradication of *H. pylori *is desirable for all infected individuals.

Azithromycin has an *in vitro *bactericidal effect against *H. pylori *[57] and this study assessed its administration in association with amoxicillin for six days. As azithromycin, omeprazole and amoxicillin are subsidized by the Brazilian federal government [39], this treatment approach could be a powerful and low-cost weapon for the treatment of these diseases in our country. It is also noteworthy the pharmacokinetic characteristics of azithromycin [58], which can provide a shorter treatment and favor the patients' compliance. Mild and tolerable adverse effects can be expected, similar to those observed with clarithromycin therapy.

Several studies obtained good efficacy in *H. pylori *eradication with short-term triple therapy using azithromycin, amoxicillin and a proton pump inhibitor [59-61]. Probably better efficacy can be achieved with a higher dose of azithromycin [33].

The present study has indeed showed high compliance and a few significant adverse effects; however, low eradication rates were observed. It is important to stress that, a macrolide use can probably decrease future treatment efficacy, when these compounds are reutilized.

Although azithromycin is a macrolide that reaches high concentration in plasma and in the gastric mucosa, the low eradication rates can be explained by its low concentration in the gastric juice [62]. It is noteworthy that a study observed an eradication rate of 80% 30 days after the treatment and 20% after 60 days, which suggests a temporary suppression of the infection [30].

## Conclusion

The short term therapy for *H. pylori *eradication using azithromycin 500 mg and omeprazole 20 mg in a single daily dose, associated with amoxicillin 500 mg three times a day resulted in few adverse effects, and high compliance with low costs. But low eradication rates observed with this approach do not allow recommending it as an alternative treatment.

## Competing interests

The authors declare that they have no competing interests.

## Authors' contributions

All authors contributed to the design of the study. Acquisition of data and quality control: FMS, JNE, ACST, TN–R. Analysis and interpretation of data: FMS, JNE. Draft of manuscript: FMS. Endoscopic examinations: RCB. Laboratory assessment: RM. All authors have read and approved the final manuscript.

## Pre-publication history

The pre-publication history for this paper can be accessed here:


